# A Printing Strategy for Embedding Conductor Paths into FFF Printed Parts

**DOI:** 10.3390/polym15173498

**Published:** 2023-08-22

**Authors:** Timo Banko, Stefan Grünwald, Rainer Kronberger, Hermann Seitz

**Affiliations:** 1 Faculty of Process Engineering, Energy and Mechanical Systems, TH Köln—University of Applied Sciences, 50679 Cologne, Germany; 2 Faculty of Information, Media and Electrical Engineering, TH Köln—University of Applied Sciences, 50679 Cologne, Germany; 3 Chair of Microfluidics, Faculty of Mechanical Engineering and Marine Technology, University of Rostock, 18059 Rostock, Germany; 4 Department Life, Light and Matter, University of Rostock, 18059 Rostock, Germany

**Keywords:** 3D printing, fused filament fabrication, multi-material printing, 3D printed electronics, printing strategy, isotropic conductive adhesive

## Abstract

A novel approach to manufacture components with integrated conductor paths involves embedding and sintering an isotropic conductive adhesive (ICA) during fused filament fabrication (FFF). However, the molten plastic is deposited directly onto the adhesive path which causes an inhomogeneous displacement of the uncured ICA. This paper presents a 3D printing strategy to achieve a homogeneous cross-section of the conductor path. The approach involves embedding the ICA into a printed groove and sealing it with a wide extruded plastic strand. Three parameter studies are conducted to obtain a consistent cavity for uniform formation of the ICA path. Specimens made of polylactic acid (PLA) with embedded ICA paths are printed and evaluated. The optimal parameters include a groove printed with a layer height of 0.1 mm, depth of 0.4 mm, and sealed with a PLA strand of 700 µm diameter. This resulted in a conductor path with a homogeneous cross-section, measuring 660 µm ± 22 µm in width (relative standard deviation: 3.3%) and a cross-sectional area of 0.108 mm^2^ ± 0.008 mm^2^ (relative standard deviation 7.2%). This is the first study to demonstrate the successful implementation of a printing strategy for embedding conductive traces with a homogeneous cross-sectional area in FFF 3D printing.

## 1. Introduction

In the context of Industry 4.0 and the Internet of Things (IoT), components and assemblies with integrated electrical circuits are becoming deeply significant [1]. The integration of conductive structures in additive manufactured components has the potential to transform them into “smart devices”, for example, by printing antennas directly into the housing [2], printing circuits onto drones [3] or 3D printing triboelectric nanogenerators (TENGs) for energy harvesting [4]. Many research works have been conducted in the field of 3D printed electronics in recent years [5,6,7,8]. In general, the integration of conductive structures is possible in various additive manufacturing (AM) technologies such as binder jetting [9], vat photopolymerization [10,11] and material extrusion [12,13,14]. The material extrusion process is particularly suitable, because all common plastics are good insulators. In contrast to binder jetting or vat photopolymerization processes, the printing surface is free of interfering powders or liquids [6,14]. In recent years, research activities have focused on the integration of electrically conductive inks, pastes, or adhesives into 3D printing using the direct ink writing (DIW) method [12,13,14,15,16,17]. In order to develop their electrical conductivity, a post-treatment of the conductor path or the entire component is necessary, which is known as sintering. The most common sintering techniques are thermal sintering [18] and laser sintering [19]. However, the main drawbacks of these techniques are the long process times and the limited usability for substrates with low heat resistance [20]. For this reason, research into novel sintering strategies is desirable, for example, to embed conductive paths in substrates with low glass transition or melting temperature such as polylactic acid (PLA), which is a commonly used material in FFF printing due to its easy processability.

**Figure 1:**
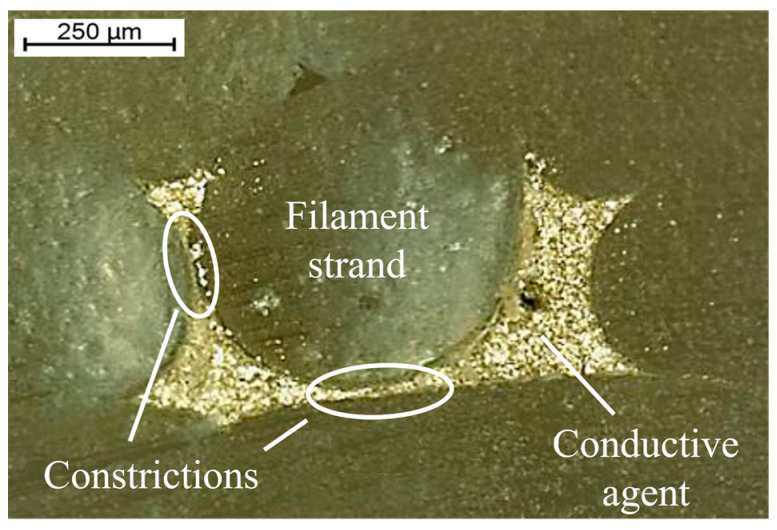
Cross-section of the embedded and highly deformed structure.

In a previous work, the possibility of embedding and sintering conductive fluids in FFF printed PLA (Filamentworld, Neu-Ulm, Germany) structures in a single process step was presented. An isotropic conductive adhesive (ICA) DELO DUALBOND^®^ IC4753 (DELO Industrie Klebstoffe GmbH & Co., KGaA, Windach, Germany) filled with silver microparticles was first dispensed in the immediate vicinity of the extruder nozzle and the molten and hot plastic were deposited directly on top [21]. However, the obtained conductive structures had an undefined cross-section due to the deformation of the ICA during plastic deposition (Figure 1). This led to an uneven resistance distribution in the conductive paths with regions of higher and lower electrical conductivity. One approach to enhance the homogeneity of the cross-section is to dispense the conductive material into milled channels. Examples of this are the 3Dn manufacturing system from nSrypt used by Arnal et al. [15] or the multi^3D^ system described by Espalin et al. [16]. However, additional components for micro-milling are not in line with the objective of embedding conductive paths in one single process step.

The integration of conductive paths into FFF printing involves two essential aspects. First, the printing strategy to embed the conductive ICA homogeneously. And secondly the curing and sintering of the ICA. This paper reports on the development of a printing strategy to embed the conductive ICA with a focus on the cross-sectional geometry of the conductor path. The aim is to determine the process parameters to achieve conductor paths with a homogeneous cross-section along its length.

## 2. Materials and Methods

### 2.1. Materials

The conductive material used is ICA DELO DUALBOND^®^ IC4753 (DELO Industrie Klebstoffe GmbH & Co., KGaA, Windach, Germany). This ICA is based on acrylate and filled with silver micro particles. The density specified in the datasheet is 3.17 g/cm^3^ [22]. According to the manufacturer’s specifications, the electrical resistivity after curing is 0.1 mOhm·cm and the viscosity amounts to 30 Pa·s at 23 °C and 10 s^−1^ shear rate [22].

White PLA (Filamentworld, Neu-Ulm, Germany) is selected as the substrate material, which has a lower density than the IC4753, i.e., 1.24 g/cm^3^ [23]. The ICA and PLA both have a shear-thinning behavior [24]. With an increasing shear rate, the viscosity decreases. In addition, the viscosity of both materials is temperature dependent.

### 2.2. FFF Printer Customization

**Figure 2:**
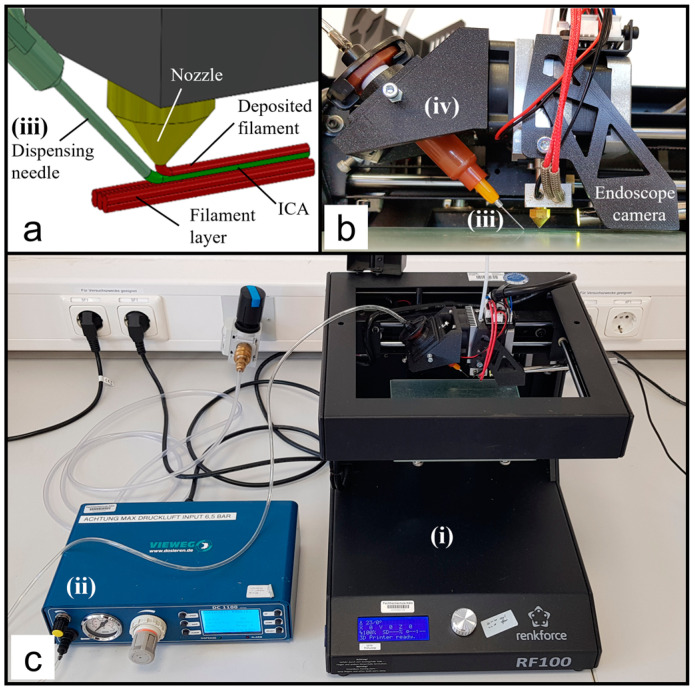
Modified 3D printing set-up: (**a**) Schematic close-up of the printing process; (**b**) print head and custom holder for the dispensing system; (**c**) digital dispenser and the RF100 FFF printer.

Liquid (conductive) materials can be applied through various methods, for example, contactless methods such as ink-jetting or aerosol jetting [25]. However, the contactless dosing of particle-filled fluids such as ICA is complex and special dosing valves are required. Because of this, the DIW method is used to apply the ICA. The DIW method can be easily integrated with already available desktop FFF printers. The experimental setup is shown in Figure 2 and consists of four key components:

(i) A Cartesian XY-head, single extruder FFF printer (RF100 by Renkforce, Hirschau, Germany). The printhead moves along a X and Y gantry. The distance of the nozzle to the non-heated build platform is set by the movement of the print bed in Z direction.
(ii) A digital dispenser (model DC1100 by VIEWEG GmbH, Kranzberg, Germany) for dosing the ICA using the time-pressure technique.
(iii) A 5 cc syringe barrel and a dispensing tip with an inner cannula diameter of 0.34 mm (GONANO Dosiertechnik GmbH, Breitstetten, Germany), and both are lightproof against UV light.
(iv) A 3D-printed holder to attach the dispensing system to the print head. The dispensing needle is aligned with the nozzle by four thrust screws. The ICA is applied in front of the plastic deposition. Due to this arrangement, the embedding of the ICA is done linearly from right to left (Figure 2a,b). Since the dispensing needle is 1 mm lower than the nozzle, the syringe cartridge with the dispensing needle is only inserted while the ICA is being deposited.

An endoscope camera (ASIN: B07XL9VTZ1, Amazon EU S.à r.l., Munich, Germany) was used to visualize the embedding process and to monitor the plastic deposition, e.g., to detect leakage of the ICA during embedding. Specimens were designed in the computer-aided design (CAD) software SolidWorks (2020 SP5.0) (Dassault Systèmes SolidWorks Corp., Vélizy-Villacoublay, France) and converted to a Standard Triangulation Language (STL) file. From this, g-codes were generated using the slicer software Simplify3D (v4.1.2) (Cincinnati, OH, USA), in which the printing parameters can be modified layer by layer.

### 2.3. Process Parameters

**Figure 3:**
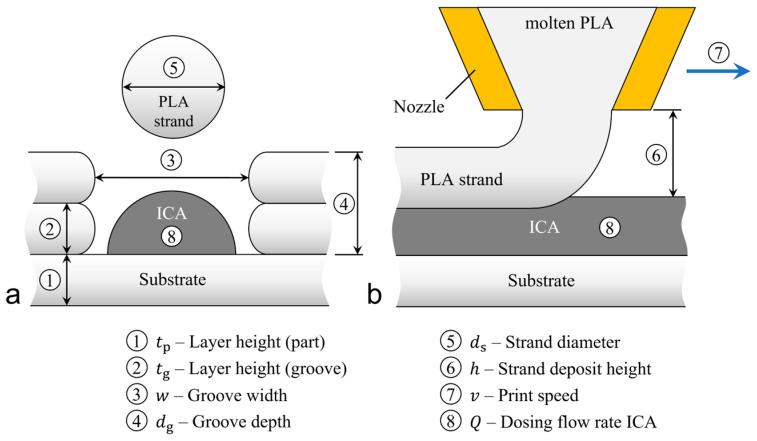
Parameters of the embedding process: (**a**) Schematic representation of the process in cross-section; (**b**) a side view.

The new 3D printing strategy is to embed the ICA in a printed groove and seal it with a wide single plastic strand. In FFF printing, and especially in the process described, there is a variety of parameters which influence the embedding process and the geometry of the conductor paths. Figure 3 shows the embedding process in cross-section and side view with the parameters of the process. Namely, these are the layer heights during 3D printing of the part and consequently during the realization of the groove, the groove dimensions, the amount of plastic, the deposition height to seal the groove, the print speed, and the amount of applied ICA.

During plastic deposition, extrusion forces are generated that act on the already printed layers. In the ordinary (multi-material) FFF process, the underlying layers are already cured, and the forces have no significant influence on them. In the new printing strategy, however, the ICA is not yet cured at the time of plastic deposition. The viscosity of the IC4753 stated in the datasheet is 30 Pa·s [22]. The zero viscosity of PLA ranges between 9000 Pa·s (at 180 °C) and 1000 Pa·s (at 230 °C) [24]. Owing to these differences in viscosity, deformation of the conductor path cannot be avoided completely. Embedding of the ICA in the groove is intended to restrict lateral deformation of the ICA.

The extrusion forces acting normally to the deposited layer are the weight force caused by the weight of the molten plastic, the impulse force caused by the molten plastic decelerating, and the force caused by the interaction of the nozzle with the deposited plastic when the nozzle compresses the strand [26]. Plott et al. [26] measured these forces that occurred during extrusion in additive manufacturing (AM). They found that the interaction of the nozzle with the deposited material was the major force component [26]. This force can be minimized by adjusting the flow rate and the height of the nozzle above the ICA.

The flow rates of the plastic and the ICA can only be considered in conjunction with the printing speed. In this work, the amount of extruded plastic is described by the strand diameter $d_{s}$. The 3D printing layer height influences the overall print speed. In general, greater layer heights reduce the printing time, but also reduce the quality of the print result [27]. If necessary, the layer heights for the entire part and the groove can have different values.

### 2.4. Experimental Approach

**Figure 4:**
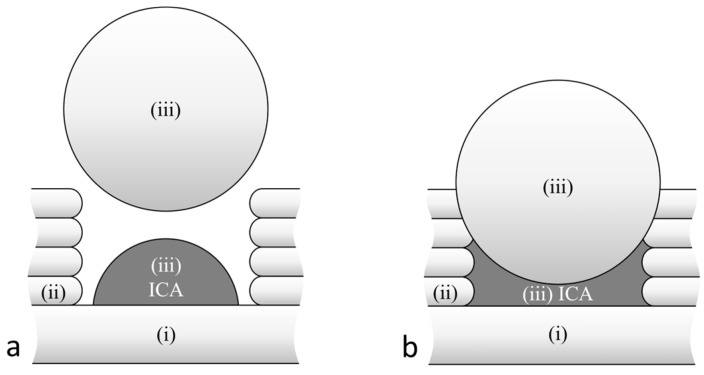
Cross-sectional representation of the embedding strategy showing the bottom layer (i), the printed groove (ii), and the ICA embedded through the sealing strand (iii): (**a**) After dispensing the adhesive and before depositing the filament strand; (**b**) after embedding the adhesive through the filament strand.

The 3D printing strategy pursued consisted of three process steps, as shown in Figure 4. In the first step, the substrate is printed up to the layer on which the ICA will be embedded (Figure 4(i)). Then, the groove is printed with a specific width, depth, and layer height (Figure 4(ii)). In this groove, the ICA will be inserted and sealed with the wide molten plastic strand (Figure 4(iii)). The dimensions of the groove and the strand diameter are matched so that the sides of the groove support the plastic strand. In this way, the extrusion forces are dissipated through the component. A defined cavity is created into which the ICA is moulded. This cavity consequently determines the cross-sectional geometry of the conductor path.

In order to realize the described embedding strategy, three parameter studies were carried out in sequential order. The results of each study are included as fixed parameters in the next study. First, the layer height of the groove is investigated with regard to the flatness of the groove sides and the dimensional accuracy of the groove. Second, the strand diameter is varied so that the strand seals the groove. And third, the groove depth is increased until a defined cavity is formed. Finally, specimens with embedded ICA are printed with the determined parameters to evaluate the strategy. Table 1 lists the basic print parameters defined for the parameter studies described. The selected values for nozzle diameter, nozzle temperature, layer height, and print speed are typical values that are often used for printing PLA. The remaining parameters are defined by or result from physical boundary conditions. For example, the width of the groove must be larger than the outer diameter of the dosing needle.

The dosing parameters are listed in Table 2. Since the ICA contains silver particles, the inner diameter of the cannula is chosen to avoid clogging. The dosing speed is the same as the print speed during embedding. The required dosing flow rate Q of the ICA can be calculated from the cross-sectional area of the cavity A and the print speed v:

(1) Q=A⋅v

#### 2.4.1. Groove Layer Height

In FFF printing, the cross-section of the deposited plastic does not have a perfectly rectangular geometry. Rather, the sides of the strands are rounded and can each be approximated by a semicircle. The diameter of the semicircle corresponds to the layer height [28]. Therefore, the layer height of the printed groove influences the flatness of its sides.

**Figure 5:**
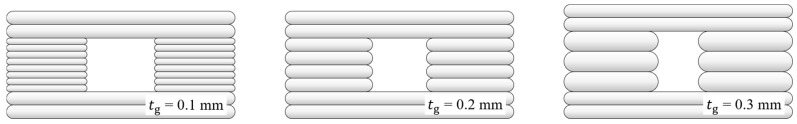
Variation of the groove layer heights.

In the embedding process described, the groove limits the lateral deformation of the ICA. Thus, the layer height influences the cross-sectional geometry of the embedded conductor path. In order to determine the layer height that leads to the most homogeneous cross-section, 40 mm long and 3.8 mm wide specimens with 600 µm wide grooves without the sealing strand were printed. The layer height of the groove is varied between 0.1 mm (8 layers), 0.2 mm (4 layers) and 0.3 mm (3 layers), with two 0.2 mm thick layers each as bottom and top layers (Figure 5). The printed specimens are cut with a fine saw. The cut surfaces are smoothed with a scalpel. The geometry and the dimensional accuracy of the grooves are evaluated using a digital microscope (Leica DVM6, Leica Microsystems GmbH, Wetzlar, Germany). The determined layer height leading to the flattest sides of the groove remains unchanged for the subsequent experiments.

#### 2.4.2. Strand Diameter

**Figure 6:**
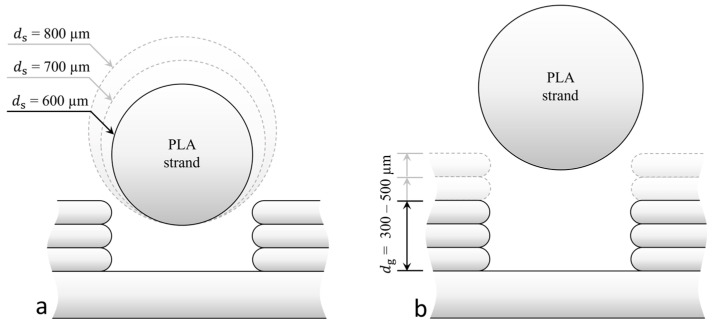
Schematic representation of: (**a**) the variation of the strand diameter; (**b**) the groove depth.

In g-code, the command for extrusion E corresponds to the position of the filament spool. E is defined as the length of filament which is pushed into the nozzle. For a desired sealing strand diameter $d_{s}$, E is calculated as follows:

(2) $$E=l_{s}\cdot \frac{d_{s}^{2}}{d_{f}^{2}}$$

where $l_{s}$ represents the length of the extruded strand and $d_{f}$ the diameter of the original input filament (1.75 mm). Three specimens with 30 mm long strands with the diameters 600 µm, 700 µm and 800 µm are printed (Figure 6a). The printed groove is 0.3 mm deep with the layer height determined in the study before. As previously described, the specimens are cut, and the cross-sections are evaluated with the digital microscope. A strand diameter is appropriate if it completely seals the groove but does not take up too much volume, and thus affect the layers above.

#### 2.4.3. Groove Depth

To ensure that the extrusion forces are dissipated via the groove sides and do not act on the ICA, a cavity must be formed after strand deposition. For that purpose, three specimens with a varying groove depth between 300 µm, 400 µm and 500 µm were printed, as seen in Figure 6b. The cross-sections of the specimens were prepared and evaluated using a digital microscope as previously described.

#### 2.4.4. Groove Sealing Strategy Evaluation

**Figure 7:**
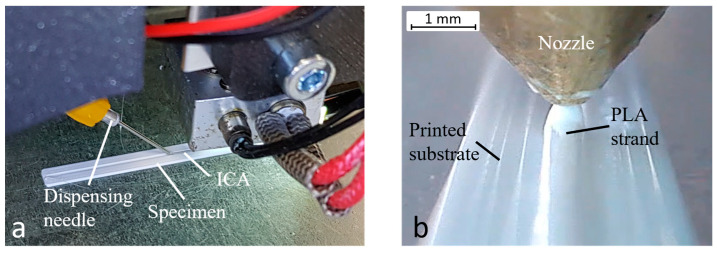
Fabrication of the specimens with embedded adhesive path (**a**). Embedding process seen through an endoscope camera (**b**).

With the determined groove layer height, strand diameter, and groove depth, five specimens with embedded ICA were printed. The fabrication of a specimen is shown in Figure 7. In order to print the specimens, the dosing flow rate Q is calculated according to Equation (1). The cross-sectional area of the cavity A is determined under the digital microscope using an unfilled specimen from the tests described previously.

**Figure 8:**
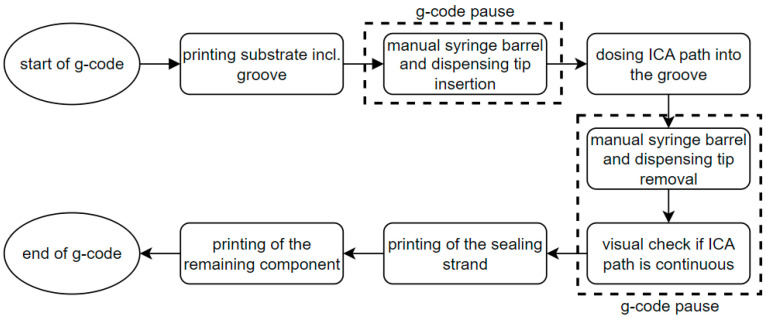
Flow chart representation of the overall printing process.

The g-code commands of the embedding process are written manually and added to the code generated by the slicer software. The schematic of the overall printing process is shown in Figure 8 and can be divided in three processes. Firstly, printing the substrate until the groove is finished. Secondly, the application of the adhesive path. And finally, the sealing of the groove and the completion of the specimen. Since dispensing is triggered manually on the digital dispenser and the syringe barrel must be removed during plastic deposition, pauses between the processes are programmed in the g-code. The fabrication of one specimen takes about 5 min. Every specimen is cut in three positions at 10 mm intervals. Each of the three cross-sectional surfaces is flattened with a scalpel for microscopy. With the digital microscope, the thickness of the conductor path at its thinnest point, its width, and its cross-sectional area are measured. In total, 15 cross-sections are evaluated.

## 3. Results and Discussion

### 3.1. Groove Layer Height

**Figure 9:**
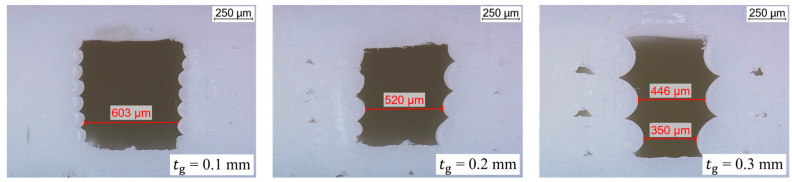
Microscope images of the cross-sections with three different groove layer heights.

Microscope images of the groove cross-sections are presented in Figure 9. As expected, the groove sides are the smoothest at a layer height of 0.1 mm. Interestingly, regarding the cross-sections, a correlation was found between the measured groove width and the layer height. With a groove layer height of 0.1 mm, the measured groove width of 603 µm corresponds almost exactly to the specified width of 600 µm. The dimensional accuracy decreases with increasing layer height. The groove width was measured to be 520 µm at $t_{g}$ = 0.2 mm and approximately 400 µm at $t_{g}$ = 0.3 mm. These results are in line with the findings of Abas et al. [29] and Deswal et al. [30]. The print time increases with decreasing layer height [27]. However, a high dimensional accuracy is required for the embedding strategy. Therefore, 0.1 mm was chosen for the groove layer height.

### 3.2. Strand Diameter

**Figure 10:**
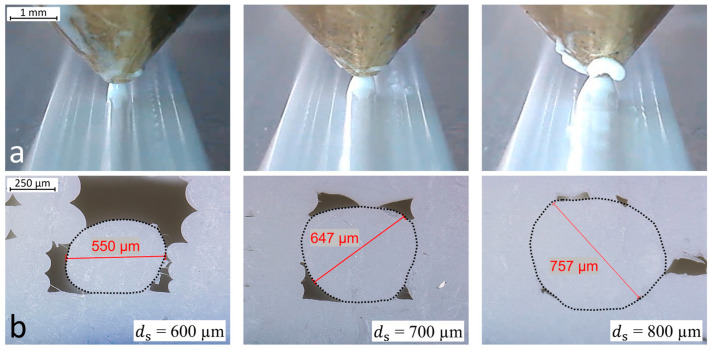
Variation of the diameter of the groove sealing strand during fabrication (**a**) and in cross-section (**b**). The boundaries between the grooves and PLA strands are visualized with dashed lines.

Deposition of the plastic strand and the resulting cross-sections under variation of $d_{s}$ are presented in Figure 10. It can be observed that the plastic strand widens just after leaving the nozzle. However, the measured strand diameters deviate from the calculated nominal values. With 550 µm instead of 600 µm and 650 µm instead of 700 µm, the sealing strand is approximately 50 µm thinner than calculated. This was found to be due to the diameter of the filament $d_{f}$ deviating from the nominal value given. Measurements with a micrometer screw gauge revealed that the input filament has an actual average diameter of 1.7 mm instead of the specified 1.75 mm. Consequently, the strand with $d_{s}$ = 600 µm is thinner than the groove width and sinks to the bottom without touching the groove sides. A groove sealing effect can be observed at $d_{s}$ = 700 µm. In the cross-section, it can be observed that the strand bonds with the plastic on the sides of the groove. At $d_{s}$ = 800 µm, so much plastic is extruded that it almost completely fills the groove. The endoscope camera images show that the strand builds up high above the print surface and takes up a lot of volume. This results in the strand negatively affecting the layers to be printed above; $d_{s}$ = 600 µm is too small and $d_{s}$ = 800 µm builds up too high. The strand with $d_{s}$ = 700 µm seals the groove without taking up too much volume; and therefore, it was chosen for the embedding strategy.

### 3.3. Groove Depth

**Figure 11:**
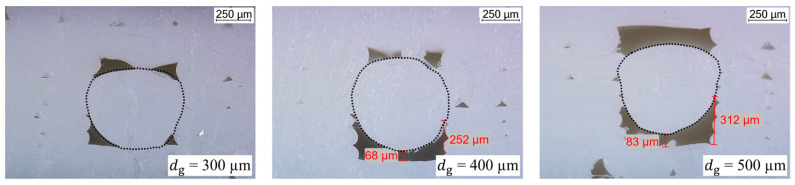
Cross-sections under variation of the groove depth. The boundaries between the grooves and PLA strands are visualized with dashed lines.

Specimens for the investigation of the groove depth were printed with $d_{s}$ = 700 µm and $t_{g}$ = 0.1 mm, according to the results of the described parameter studies. The resulting cross-sections are shown in Figure 11. With a groove depth of 300 µm, the distance between the upper edge of the groove and its bottom is too small, causing the plastic strand to touch the bottom. From $d_{g}$ = 400 µm onwards, a continuous cavity forms underneath the plastic strand. This cavity measures about 70 µm at its thinnest point in the middle. At its sides, the cavity is thicker, at about 250 µm. Increasing the groove depth to 500 µm also increases the size of the cavity, although not to the same extent. At its thinnest point, a thickness of 80 µm was measured. Towards the sides of the groove, the thickness of the cavity increases to approximately 300 µm. Consequently, by varying the groove depth, the size of the cavity and thus the cross-sectional area of the conductor path can be adjusted. However, regarding the curing and sintering of the ICA thinner conductor paths are preferable because less material needs to be sintered; and therefore, less energy is required. To investigate the homogeneity of the smallest conductor path printable with this strategy, $d_{g}$ = 400 µm was chosen for further evaluation.

### 3.4. Groove Sealing Strategy Evaluation

Equation (1) is used to determine the dosing flow rate of the ICA. The cross-sectional area of the cavity A is determined using the printed specimen with the parameters $d_{s}$ = 700 µm, $t_{g}$ = 0.1 mm, and $d_{g}$ = 400 µm. A amounts to 0.10 mm^2^. With the defined print speed during embedding of 300 mm/min, the required dosing flow rate is 30 µL/min.

**Figure 12:**
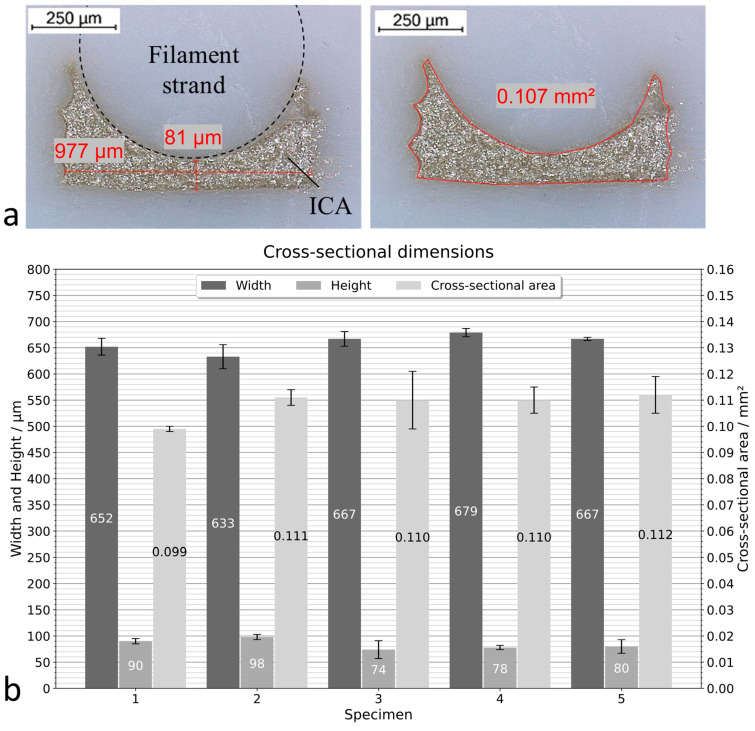
Measured width, height, and cross-sectional area of the embedded ICA paths: (**a**) Microscopic evaluation of one cross-section; (**b**) average values including standard deviations for the width, height, and cross-sectional area of the ICA paths of all specimens.

A microscopic evaluation of the cross-sections shows that the ICA was completely embedded (Figure 12a). All adhesive paths were continuous after visual inspection before the sealing strand was deposited and no capillary effects were observed. No leakage of excess adhesive from the groove was noticed during fabrication. The average values including standard deviations (SD) for the width, height, and cross-sectional area of the ICA paths are shown in Figure 12b. The mean width of the conductor paths of all specimens is 660 µm ± 22 µm. The relative standard deviation (RSD) is relatively low at 3.3%. For the individual specimens, the SDs of the widths are in the range from 3 to 23 µm, which corresponds to RSDs from 0.5 to 3.7%. The thickness of the ICA traces measured at the thinnest points averages 84 µm with a comparatively large SD of 13.6 µm (RSD = 16.2%). These deviations result mainly from Specimens 3 (SD = 12.8 µm/RSD = 16%) and 5 (SD = 17.3 µm/RSD = 23.5%). In contrast, the SDs of the thicknesses of Specimens 1, 2, and 4 range between 3.6 µm (RSD = 4.6%) and 5.4 µm (RSD = 5.7%). The cross-sectional area is directly related to its width and thickness. For this reason, larger deviations are observed in Specimens 3 and 5. The RSDs of the cross-sectional areas of these specimens deviate between 6.1% and 9.7. The RSD of the other specimens ranges between 0.8 and 4.8%. Overall, the embedded ICA paths have a mean cross-sectional area of 0.108 mm^2^ with a total SD of 0.008 mm^2^ (RSD = 7.2%).

The results show that embedding of ICA paths with a homogeneous cross-section along its length is possible with the presented printing strategy based on groove sealing and the determined parameters. The minor variations in the width of the embedded path are in line with the observed dimensional accuracy of the groove printed with a 0.1 mm layer height. Compared to Espalin et al. [16], the findings show that it is not necessary to micro mill the groove to achieve a conductor path width accuracy of less than 4%. The thickness of the embedded structure is significantly dependent on the height at which the plastic strand forms a bond with the sides of the groove. The influencing parameters are the extrusion flow rate and the width of the printed groove. The homogeneity of the conductor paths is of great importance for homogeneous electrical conductivity. The conductor paths achieved suggest that there should be no peaks in electrical resistance due to variations in cross-sectional areas or constrictions. The cross-sectional areas of the ICA paths correspond to those of the measured cavity. This indicates that the extrusion forces are dissipated through the sides of the groove. Therefore, the nature of the medium to be embedded should be of secondary importance for the geometry of the conductor path and only need to be considered for the dosing process. With the presented strategy, thus, it should be possible to embed media with lower viscosity than that of the ICA.

## 4. Conclusions

A novel printing strategy for embedding conductor paths into 3D printed parts was presented. It was successfully demonstrated that the groove sealing strategy can be used to embed conductor paths made of ICA in FFF printed parts with homogeneous cross-sections in one process step. A groove layer height of 0.1 mm results in the flattest groove sides and the highest dimensional accuracy of the groove width. With a sealing strand diameter of 700 µm, a groove width of 600 µm, and a groove depth of 400 µm, a defined cavity is created in which the ICA path is uniformly formed. The conductor paths printed with the described parameters have a mean cross-sectional area of 0.108 mm^2^ with a relative standard deviation of 7.2%. The mean width of the paths is 660 µm with a relative standard deviation of 3.3%.

In the present study, the electrical properties of the conductors were not investigated. In order to be able to undertake these investigations, the next step must focus on the thermal post-processing of the ICA, i.e., the curing and sintering of the ICA. The sealing strand is deposited directly onto the conductive liquid, and both share a considerable large contact area. Therefore, it should be investigated whether sufficient heat transfer occurs between the sealing strand and the conductor path during FFF 3D printing to cure and sinter the conductive fluid so that high electrical conductivity is achieved. Further sintering methods which could be considered are non-selective methods such as thermal sintering and intense pulsed light sintering or selective sintering methods such as laser sintering or ohmic sintering. In addition, the investigations were limited to the realization of simple unidirectional conductor paths. Technical extensions and experimental investigations are also required on this point in order to be able to realize two-dimensional or even three-dimensional conductor paths in complex 3D printed components.

## Figures and Tables

**Table 1:** Defined FFF print parameters for PLA.

Parameter	Value
Nozzle diameter	0.4 mm
Nozzle temperature	210 °C
3D printing layer height *t*_p_ (part)	0.2 mm
Groove width *w*	600 µm
Strand deposit height *h* (Above top edge of the groove)	$d_{s}$—100 µm
Print speed (during printing)	3000 mm/min
Print speed (during embedding) v	300 mm/min

**Table 2:** Defined dosing parameters for ICA.

Parameter	Value
Cannula inner diameter	0.34 mm
Cannula length	½ in
Dosing speed	300 mm/min
Dosing flow rate Q	A⋅v

## Data Availability

The data presented in this study are available on request from the corresponding author. The data are not publicly available due to the confidentiality of the running project.
